# Risk of intensive care unit admission and mortality in patients hospitalized due to influenza A or B and SARS‑CoV‑2 variants Omicron or Delta

**DOI:** 10.1002/iid3.1269

**Published:** 2024-07-05

**Authors:** Omid Rezahosseini, Casper Roed, Adin Sejdic, Mads Frederik Eiberg, Lene Nielsen, Jonas Boel, Caroline Klint Johannesen, Maarten van Wijhe, Kristina Træholt Franck, Sisse Rye Ostrowski, Birgitte Lindegaard, Thea K. Fischer, Troels Bygum Knudsen, Jon Gitz Holler, Zitta Barrella Harboe, the COVID‐19 Omicron Delta study group collaborators, Betina Lindgaard‐Jensen, Christian Søborg, Thyge Lynghøj Nielsen, Peter Haahr Bernhard, Emilie Marie Juelstorp Pedersen, Gertrud Baunbæk Egelund, Inger Hee Mabuza Mathiesen, Naja Zenius Jespersen, Pelle Trier Petersen, Hans Eric Sebastian Seitz‐Rasmussen, Barbara Bonnesen Bertelsen, Morten Bestle, Henrik Andersen, Thomas Ulrik Skram, Sarah Altaraihi, Pradeesh Sivapalan, Jens‐Ulrik Stæhr Jensen, Kristian Bagge, Kristina Melbardis Jørgensen, Magnus Glindvad Ahlström, Sofie Rytter, Nina le Dous, Pernille Ravn, Nanna Reiter, Daria Podlekareva, Jesper Andreas Knudsen, Lars‐Erik Kristensen, Cæcilie Leding, Thomas Benfield, Ole Kirk, Sigurdur Thor Sigurdsson, Martin Schou Pedersen

**Affiliations:** ^1^ Department of Pulmonary and Infectious Diseases Copenhagen University Hospital, North Zealand Hillerød Denmark; ^2^ Department of Clinical Medicine, Faculty of Health and Medical Sciences University of Copenhagen Copenhagen Denmark; ^3^ Department of Science and Environment, PandemiX Center Roskilde University Roskilde Denmark; ^4^ Department of Clinical Microbiology Copenhagen University Hospital, Herlev & Gentofte Greater Copenhagen Denmark; ^5^ Department of Clinical Research Copenhagen University Hospital, North Zealand Denmark; ^6^ Statens Serum Institut Copenhagen Denmark; ^7^ Department of Clinical Immunology Copenhagen University Hospital—Rigshospitalet Copenhagen Denmark; ^8^ Department of Public Health, Faculty of Health and Medical Sciences University of Copenhagen Copenhagen Denmark; ^9^ Department of Intensive Care Copenhagen University Hospital, North Zealand Hillerød Denmark; ^10^ Department of Medicine, Section of Respiratory Medicine Copenhagen University Hospital ‐ Herlev and Gentofte Hospital Greater Copenhagen Denmark; ^11^ Department of Clinical Microbiology Copenhagen University Hospital, Amager‐Hvidovre Hospital Greater Copenhagen Denmark; ^12^ Department of Clinical Microbiology Copenhagen University Hospital, Herlev‐Gentofte Hospital Greater Copenhagen Denmark; ^13^ Department of Internal Medicine, Section for Infectious Diseases Copenhagen University Hospital, Herlev‐Gentofte Hospital Greater Copenhagen Denmark; ^14^ Department of Intensive Care Copenhagen University Hospital, Bispebjerg‐Frederiksberg Hospital Greater Copenhagen Denmark; ^15^ Department of Respiratory Medicine and Infectious Diseases Copenhagen University Hospital, Bispebjerg‐Frederiksberg Hospital Greater Copenhagen Denmark; ^16^ Parker Institut, Copenhagen University Hospital, Bispebjerg‐Frederiksberg Hospital Greater Copenhagen Denmark; ^17^ Department of Infectious Diseases Copenhagen University Hospital, Amager‐Hvidovre Hospital Greater Copenhagen Denmark; ^18^ Department of Infectious Diseases Copenhagen University Hospital, Rigshospitalet Copenhagen Denmark; ^19^ Department of Neurointensive Care Copenhagen University Hospital, Rigshospitalet Copenhagen Denmark; ^20^ Department of Clinical Microbiology Copenhagen University Hospital, Rigshospitalet Copenhagen Denmark

**Keywords:** influenza A, influenza B, intensive care units, mortality, SARS‐CoV‐2 Delta variants, SARS‐CoV‐2 Omicron variant

## Abstract

**Background:**

Respiratory viral infections have significant global health impacts. We compared 30‐day intensive care unit (ICU) admission and all‐cause mortality risks in patients with severe acute respiratory syndrome coronavirus 2 (SARS‐CoV‐2) Delta and Omicron variants versus influenza A and B (A/B).

**Methods:**

Data from two retrospective inpatient cohorts in Copenhagen were analyzed. Cohorts included hospitalized influenza A/B patients (2017–2018) and SARS‐CoV‐2 Delta/Omicron patients (2021–2022), aged ≥18 years, admitted within 14 days of a positive real‐time polymerase chain reaction test result. Cumulative ICU admission and mortality rates were estimated using the Aalen–Johansen estimator. Cox regression models calculated hazard ratios (HRs) for ICU admission and mortality.

**Results:**

The study encompassed 1459 inpatients (Delta: 49%; Omicron: 26%; influenza A: 6.4%; and influenza B: 18%). Cumulative incidence of ICU admission was 11%, 4.0%, 7.5%, and 4.1%, for Delta, Omicron, influenza A, and B, respectively. For ICU admission, adjusted HRs (aHRs) were 3.1 (*p* < .001) and 1.5 (*p* = .34) for Delta and Omicron versus influenza B, and 1.5 (*p* = .36) and 0.71 (*p* = .48) versus influenza A. For mortality, aHRs were 3.8 (*p* < .001) and 3.4 (*p* < .001) for Delta and Omicron versus influenza B, and 2.1 (*p* = .04) and 1.9 (*p* = .11) versus influenza A.

**Conclusion:**

Delta but not Omicron inpatients had an increased risk for ICU admission compared to influenza B; however, both variants were associated with higher risks of mortality than influenza B. Only Delta inpatients had a higher risk of mortality than influenza A inpatients.

## Graphical abstract

This study compared the risks of intensive care unit (ICU) admission and mortality among patients with severe acute respiratory syndrome coronavirus 2 Delta and Omicron variants versus those with influenza A and B. It found that patients with the Delta variant had a higher risk of ICU admission and mortality compared to those with influenza B, and a higher mortality risk than those with influenza A. However, while Omicron patients also had a higher mortality risk than influenza B patients, they did not show an increased risk for ICU admission compared to influenza B.

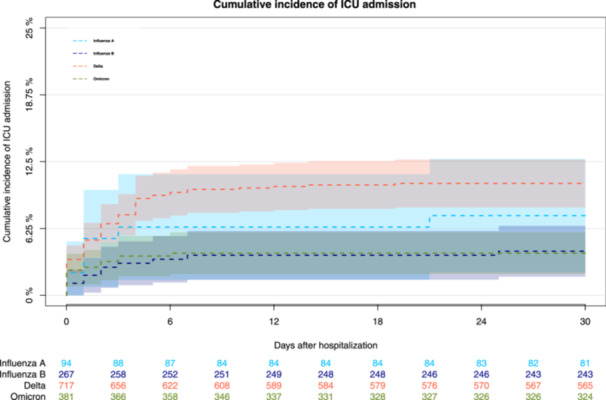

## 1 BACKGROUND

Respiratory viral infections pose significant global health challenges, impacting millions of individuals annually.^1^ More than a century ago, between 1918 and 1920, an influenza A (H1N1) pandemic affected over one‐third of the world's population, resulting in an estimated 20−50 million deaths.^2^ The influenza pandemic was neither the first nor the last in a series of calamities for which respiratory viral infections were responsible. In December 2019, the severe acute respiratory syndrome coronavirus 2 (SARS‐CoV‐2) pandemic emerged, leading to over 769 million infections and more than 6.9 million deaths worldwide by August 2023.^3^

Fortunately, the advent of vaccines has provided an effective strategy to prevent or mitigate the severity of influenza and SARS‐CoV‐2 infections.^4^, ^5^ However, the constant evolution of new viral strains and variants has proved a significant obstacle in controlling these infections.^6^ Despite successful vaccination programs, new SARS‐CoV‐2 variants continue to circulate and seasonal influenza claims the lives of over 650,000 people annually.^7^

Patients with severe influenza or coronavirus disease 2019 (COVID‐19) often require hospitalization, and past data indicate that more than one‐third of these severe cases require intensive care or result in fatality following admission.^8^, ^9^ Whereas influenza epidemics have been studied extensively, COVID‐19 is a relatively new and emerging disease. It has been shown that the risk of intensive care unit (ICU) admission and mortality varies among different SARS‐CoV‐2 variants.^10^, ^11^ Moreover, several studies have indicated that adult patients with influenza A may face a greater risk of severe outcomes, including ICU admission and mortality, than patients affected by influenza B.^12^, ^13^ However, contrasting findings have been reported by other studies.^14^, ^15^, ^16^ Thus, we assessed the risk of ICU admission and all‐cause mortality due to the SARS‐CoV‐2 variants Delta and Omicron, compared with influenza A/B, within 30 days after hospitalization.

## 2 METHODS

### 2.1 Study design and setting

We employed data from two retrospective inpatient cohort studies conducted within the Capital Region of Copenhagen, Denmark, which will be described below. The Capital Region of Copenhagen encompasses a population of approximately 1.8 million individuals. The region is responsible for administering and coordinating healthcare services within its boundaries. It operates several major hospitals and medical facilities.^17^

### 2.2 The cohort of patients with influenza A/B

Nordsjællands Hospital (NOH) is one of the university hospitals in the Capital Region, which provides nonstop medical services to approximately 323,000 inhabitants in North Zealand. The NOH emergency department evaluates/treats approximately 85,000 patients annually. All symptomatic patients were screened for influenza at the emergency department and had nasopharyngeal swabs collected for analysis via real‐time polymerase chain reaction (RT‐PCR) using the Prodesse ProFlu™+ (Hologic, Inc.) assay procedure to identify the influenza A/B viruses and Respiratory Syncytial Virus. Patients with a positive RT‐PCR test result for influenza A or influenza B were distinguished by further testing at the Department of Clinical Microbiology at Herlev Hospital, Denmark. The administrative database at NOH was cross‐checked to confirm the hospitalizations.

### 2.3 The cohort of patients with SARS‐CoV‐2 variants Delta and Omicron

In Denmark, Delta was the predominant SARS‐CoV‐2 variant from July to December 2021, with the first cases of the Omicron variant detected in November 2021.^18^, ^19^ Omicron cases subsequently peaked in mid‐January 2022.^18^, ^19^ Therefore, all individuals who tested positive for SARS‐CoV‐2 via RT‐PCR and had a hospital contact between September 1, 2021 and February 11, 2022 were eligible for inclusion. The cohort characteristics and surveillance and screening algorithms for SARS‐CoV‐2 variants in Denmark have been described previously.^20^ Variant identification relied on whole‐genome sequencing or variant‐specific RT‐PCR conducted at the Statens Serum Institut (SSI) or departments of clinical microbiology within the region.

### 2.4 Participants

We enrolled adult patients (≥18 years) who were hospitalized for more than 12 h within 14 days of a positive RT‐PCR test for influenza or the SARS‐CoV‐2 variants Delta or Omicron.^21^, ^22^ We excluded patients under 18 years of age, patients admitted to the emergency department for less than 12 h, patients without a valid Danish personal identification number, asymptomatic patients in whom either influenza or SARS‐CoV‐2 was incidentally found or detected as part of a screening procedure upon admission, and patients with a positive RT‐PCR influenza or SARS‐CoV‐2 test result obtained more than 48 h after hospitalization (i.e., those with presumed nosocomial infections). All patients who did not meet the exclusion criteria were included.

### 2.5 Variables and outcomes

The following predefined variables were included in the analyses: age, sex, comorbidities, receiving immunosuppressive agents, date of ICU admission, date of death, and documented vaccination(s) before hospital admission. The primary outcomes of interest were ICU admission and all‐cause mortality within 30 days after hospitalization.

### 2.6 Definitions

Comorbidities included diabetes, cardiac diseases, renal diseases, active cancer, and receiving immunosuppressive agents.

Immunosuppression was defined as treatment with more than 20 mg of prednisolone daily or an equivalent dose of other corticosteroids, treatment with biological agents, methotrexate, or chemotherapy (Supporting Information: Table 1).

Treatment was defined as receiving at least one of the following drugs: dexamethasone (6–12 mg), remdesivir, monoclonal antibodies (including sotrovimab, casirivimab/imdevimab), and IL‐6 inhibitors at standard dose for patients with SARS‐CoV‐2 and oseltamivir at standard dose for patients with influenza.

Vaccination: Patients were considered sufficiently vaccinated against influenza if they had received an influenza vaccine from September 1, 2017 to April 1, 2018, and at least 14 days before hospital admission. For COVID‐19, patients were considered sufficiently vaccinated if they had received at least two doses of the vaccine, with the second dose administered at least 14 days before hospital admission.

### 2.7 Data sources

We obtained data from various sources, including the Danish Civil Register,^23^ the National Patient Register,^24^ the Danish Vaccination Register,^25^ the Danish Microbiology Database (MIBA),^26^ and the National COVID‐19 surveillance system at the SSI.^21^, ^22^ In Denmark, each person is assigned a unique personal identification number, known as the CPR number. This number enables access to information about an individual's birth date, gender, hospitalization records, and death. It also facilitates tracking patients through the national databases mentioned. Electronic medical records were also reviewed for all unique patients who met the inclusion criteria. Experienced medical doctors and nurses conducted the review process. All extracted information was recorded in a Research Electronic Data Capture (REDCap) database.

### 2.8 Statistical analyses

We presented continuous data as medians with interquartile ranges (IQRs) and reported proportions as percentages. We employed the Mann–Whitney *U* test to compare median differences, and we utilized chi‐square tests to evaluate frequency distributions.

The index date was defined as the date of hospital admission. The patients were followed from the date of hospitalization to ICU admission, death, or Day 30 after hospitalization, whichever came first, to estimate the cumulative incidence of ICU admission and compare risks of ICU admission. To estimate the risk of mortality, the patients were followed from the date of hospitalization to either the date of death or day 30 after hospitalization, whichever came first.

We estimated the cumulative incidence of ICU admission using the Aalen–Johansen estimator, with mortality as a competing risk, and assessed statistical differences using Gray's test. We conducted survival analyses and employed Cox regression models to calculate ICU admission and mortality hazard ratios (HRs). The proportional hazard assumptions were assessed using Schoenfeld's residuals and found to be valid. The multivariable model was stratified by age group, sex, number of comorbidities, treatment, and vaccination status. We used Cox regression models twice: once with patients hospitalized with influenza B as the reference group, and a second time with those hospitalized with influenza A as the reference group. We performed statistical analyses using R software (ver. 4.1.0; R Development Core Team, 2021) and a *p* ≤ .05 was considered statistically significant.

### 2.9 Sensitivity analyses

In a sensitivity analysis, we considered influenza A and B as a single group and estimated the cumulative incidence of ICU admission and compared the risk of ICU admission and mortality 30 days after hospitalization, as previously described.

Furthermore, we conducted a matched case‐control analysis to compare the risk of ICU admission and mortality 30 days after hospitalization. Because of the limited number of patients with influenza A, the matching process was unsatisfactory; hence, we decided to exclude patients with influenza A. Therefore, cases were defined as patients with influenza B, whereas controls were patients with SARS‐CoV‐2 variants Omicron or Delta. Cases and controls were matched based on age and sex using the MatchIt package in R software (ver. 4.3.1; R Development Core Team, 2023), and the nearest neighbor matching method was used with a 1:1 ratio. It was not possible to stratify for all age groups in the matched analyses for the multivariable model. Therefore, the model was stratified by sex, number of comorbidities, treatment, and vaccination status and adjusted for age (per 10‐year increase).

## 3 RESULTS

We included a total of 1,459 hospitalized patients, consisting of 717 (49%), 381 (26%), 94 (6.4%), and 267 (18%) patients with SARS‐CoV‐2 Delta, SARS‐CoV‐2 Omicron, influenza A, and influenza B infections, respectively. A total of 766 patients (53%) were male, median age was 72 years (IQR, 54–81), and 981 patients (67%) had at least one comorbidity. Cardiac diseases, pulmonary diseases, and diabetes were the most frequent comorbidities and were reported in 693 (48%), 363 (25%), and 298 (20%) of the patients, respectively. Patient characteristics are shown in Table 1.

**Table 1:** Characteristics of hospitalized patients with influenza A/B or COVID‐19 Delta and Omicron.

Characteristics		SARS‐CoV‐2 variants (*n* = 1098)		Influenza (*n* = 361)		Total (*n* = 1459)	*p* Value^a^
		Delta (*n* = 717)	Omicron (*n* = 381)	A (*n* = 94)	B (*n* = 267)		
Male sex, *n* (%)		398 (56%)	184 (48%)	54 (57%)	130 (49%)	766 (53%)	.04
Age (years), median [interquartile range]		69 [51–80]	72 [50–83]	74 [60–82]	73 [64−83]	72 [54–81]	<.001
Age group (years)	18–44	137 (19%)	82 (22%)	9 (9.6%)	18 (6.7%)	246 (17%)	<.001
	45–64	188 (26%)	67 (18%)	21 (22%)	56 (21%)	332 (23%)	
	65–79	218 (30%)	120 (32%)	30 (32%)	98 (37%)	466 (32%)	
	≥80	174 (24%)	112 (29%)	34 (36%)	95 (36%)	415 (28%)	
Comorbidities	Diabetes	150 (21%)	71 (19%)	18 (19%)	59 (22%)	298 (20%)	.70
	Cardiac diseases	312 (44%)	171 (45%)	53 (56%)	157 (59%)	693 (48%)	<.001
	Pulmonary diseases	166 (23%)	91 (24%)	33 (35%)	73 (27%)	363 (25%)	.06
	Renal diseases	61 (8.5%)	43 (11.3%)	5 (5.3%)	16 (6.0%)	125 (8.6%)	.07
	Receiving Immunosuppressive agents^b^	77 (11%)	50 (13%)	7 (7.4%)	5 (1.9%)	139 (9.5%)	<.001
	Cancer	66 (9.2%)	60 (16%)	20 (21%)	48 (18%)	194 (13%)	<.001
Number of comorbidities	0	253 (35%)	124 (33%)	24 (26%)	77 (29%)	478 (33%)	.13
	1	233 (33%)	135 (35%)	31 (33%)	95 (36%)	494 (34%)	
	2	167 (23%)	83 (22%)	33 (35%)	74 (28%)	357 (25%)	
	≥3	64 (8.9%)	39 (10%)	6 (6.4%)	21 (7.9%)	130 (8.9%)	
Received at least one treatment, *n* (%)^c^		490 (68%)	203 (53%)	75 (80%)	195 (73%)	963 (66%)	<.001
Vaccinated, *n* (%)^d^		334 (47%)	257 (68%)	35 (37%)	92 (35%)	718 (49%)	<.001
ICU admission within 30 days, *n* (%)		75 (11%)	15 (3.9%)	7 (7.4%)	11 (4.1%)	108 (7.4%)	<.001
Death within 30 days, *n* (%)		99 (14%)	48 (13%)	9 (9.6%)	15 (5.6%)	171 (12%)	.004
Duration of hospital stay, median number of days [interquartile range]		4^2^, ^3^, ^4^, ^5^, ^6^, ^7^, ^8^, ^9^	3^1^, ^2^, ^3^, ^4^, ^5^, ^6^	4^2^, ^3^, ^4^, ^5^, ^6^, ^7^, ^8^	3^1^, ^2^, ^3^, ^4^, ^5^, ^6^	4^1^, ^2^, ^3^, ^4^, ^5^, ^6^, ^7^, ^8^	<.001

Abbreviations: COVID‐19, coronavirus disease 2019; ICU, intensive care unit; SARS‐CoV2, severe acute respiratory syndrome coronavirus 2.

^a^ We employed the Mann–Whitney *U* test to compare median differences, and we utilized chi‐square tests to analyze frequency distributions.

b The following drugs were considered immunosuppressive: >20 mg prednisolone/daily or equivalent, chemotherapy, biologicals, and methotrexate.

c Dexamethasone, remdesivir, monoclonal antibodies, and IL‐6 inhibitors for patients with SARS‐CoV‐2 versus oseltamivir for patients with influenza.

d Patients were considered sufficiently vaccinated against influenza if they received an influenza vaccine from September 1, 2017 to April 1, 2018, and at least 14 days before hospital admission. For COVID‐19, patients were considered sufficiently vaccinated if they had received at least two doses, with the second dose administered at least 14 days before hospital admission.

### 3.1 Cumulative incidence of ICU admission

**Figure 1:**
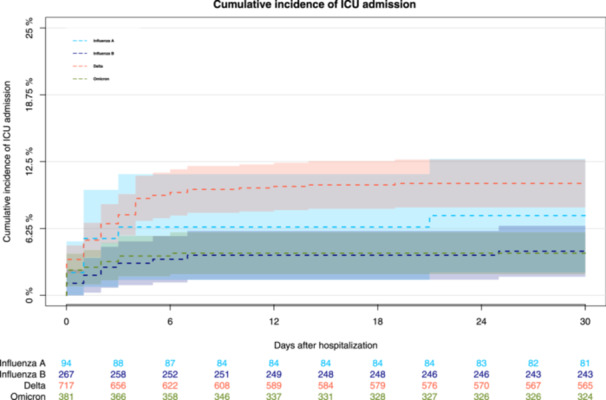
Cumulative incidence of ICU admission within 30 days after hospitalization. The cumulative incidence of ICU admission at day 30 after hospitalization was 11% (95% confidence interval [CI], 8.2–13%), 4.0% (95% CI, 2.0–5.9%), 7.5% (95% CI, 2.1–13%), and 4.1% (95% CI, 1.7–6.5%) for patients with SARS‐CoV‐2 Delta, SARS‐CoV‐2 Omicron, influenza A, and influenza B infections, respectively (*p* < .001).

The cumulative incidence of ICU admission at day 30 after hospitalization was 11% (95% confidence interval [CI], 8.2–13%), 4.0% (95% CI, 2.0–5.9%), 7.5% (95% CI, 2.1–13%), and 4.1% (95% CI, 1.7–6.5%) for patients with SARS‐CoV‐2 Delta, SARS‐CoV‐2 Omicron, influenza A, and influenza B infections, respectively (*p* < .001; Figure 1).

### 3.2 Risk of ICU admission within 30 days after hospitalization

We performed a univariable analysis using the Cox proportional hazards model. When patients with influenza B were used as the reference group, patients with SARS‐CoV‐2 Delta, SARS‐CoV‐2 Omicron, and influenza A had HRs for ICU admission of 2.7 (95% CI, 1.4–5.0; *p* = .003), 0.97 (95% CI, 0.44–2.1; *p* = .93), and 1.8 (95% CI, 0.72–4.8; *p* = .21), respectively (Table 2). In the multivariable models, the corresponding HRs for ICU admission were 3.1 (95% CI, 1.6–6.0; *p* < .001), 1.5 (95% CI, 0.66–3.4; *p* = .34), and 2.1 (95% CI, 0.78–5.7; *p* = .14), respectively. When patients with influenza A were used as the reference group, those with SARS‐CoV‐2 Delta and SARS‐CoV‐2 Omicron had adjusted HRs (aHRs) for ICU admission of 1.5 (95% CI, 0.65–3.3; *p* = .36) and 0.71 (95% CI, 0.28–1.8; *p* = 0.), 48, respectively (Supporting Information: Tables 2‐3) (Table 3).

**Table 2:** Risk of ICU admission within 30 days after hospitalization for patients with influenza A/B or COVID‐19 Delta and Omicron.

		Univariable model		Multivariable model^a^	
		Hazard ratio (95% CI)	*p* Value	Hazard ratio (95% CI)	*p* Value
Viruses	Influenza B	Ref	‐	Ref	‐
	Influenza A	1.8 (0.72–4.8)	.21	2.1 (0.78–5.7)	.14
	SARS‐CoV2‐Delta	2.7 (1.4–5.0)	.003	3.1 (1.6–6.0)	<.001
	SARS‐CoV2‐Omicron	0.97 (0.44–2.10)	.93	1.5 (0.66–3.4)	.34
Age group (years)	18–44	Ref	‐	‐	‐
	45–64	1.7 (0.90–3.2)	.10	‐	‐
	65–79	1.92 (1.06–3.5)	.03	‐	‐
	≥80	0.60 (0.29–1.3)	.18	‐	‐
Male sex		2.7 (1.8–4.1)	<.001	‐	‐
Vaccinated		0.70 (0.48–1.03)	.07	‐	‐
Received at least one treatment^b^		13 (5.3–32)	<.001	‐	‐
Number of comorbidities	0	Ref	‐	‐	‐
	1	1.3 (0.80–2.1)	.30	‐	‐
	2	1.36 (0.81–2.3)	.24	‐	‐
	≥3	1.7 (0.86–3.3)	.13	‐	‐

Abbreviations: CI, confidence interval; SARS‐CoV2, severe acute respiratory syndrome coronavirus 2.

a Model: Stratified by age group, sex, vaccination status, Tx, and number of comorbidities.

b Dexamethasone, remdesivir, monoclonal antibodies, and IL‐6 inhibitors for patients with SARS‐CoV‐2 versus oseltamivir for patients with influenza.

**Table 3:** Risk of death within 30 days after hospitalization for patients with influenza A/B or COVID‐19 Delta and Omicron.

		Univariable model		Multivariable model^a^	
		Hazard ratio (95% CI)	*p* Value	Hazard ratio (95% CI)	*p* Value
Viruses	Influenza B	Ref	‐	Ref	‐
	Influenza A	1.7 (0.76–4.0)	.19	1.8 (0.77–4.2)	.18
	SARS‐CoV2‐Delta	2.6 (1.5–4.4)	<.001	3.8 (2.1–6.7)	<.001
	SARS‐CoV2‐Omicron	2.3 (1.3–4.2)	.003	3.4 (1.8–6.2)	<.001
Age group (years)	18–44	Ref	‐	‐	‐
	45–64	2.3 (0.61–8.3)	.23	‐	‐
	65–79	12 (3.8–38)	<.001	‐	‐
	≥80	21 (6.8–67)	<.001	‐	‐
Male sex		1.3 (0.94–1.7)	.12	‐	‐
Vaccinated (Yes)		1.3 (0.95–1.7)	.10	‐	‐
Received at least one treatment^b^		2.2 (1.5–3.2)	<.001	‐	‐
Number of comorbidities	0	Ref	‐	‐	‐
	1	1.8 (1.2–2.8)	.007	‐	‐
	2	2.4 (1.6–3.8)	<.001	‐	‐
	≥3	3.1 (1.8–5.2)	<.001	‐	‐

Abbreviations: CI, confidence interval; SARS‐CoV2, severe acute respiratory syndrome coronavirus 2.

a Model: Stratified by age group, sex, vaccination status, treatment, and number of comorbidities.

b Dexamethasone, remdesivir, monoclonal antibodies, and IL‐6 inhibitors for patients with SARS‐CoV‐2 versus oseltamivir for patients with influenza.

### 3.3 Risk of mortality within 30 days after hospitalization

When patients with influenza B were used as the reference group, patients with SARS‐CoV‐2 Delta, SARS‐CoV‐2 Omicron, and influenza A had HRs for mortality of 2.6 (95% CI, 1.5–4.4; *p* < .001), 2.3 (95% CI, 1.3–4.2; *p* = .003), and 1.7 (95% CI, 0.76–4.0; *p* = .19), respectively. In the multivariable models, the corresponding HRs were 3.8 (95% CI, 2.1–6.7; *p* < .001), 3.4 (95% CI, 1.8–6.2; *p* < .001), and 1.8 (95% CI, 0.77–4.2; *p* = .18), respectively. When patients with influenza A were used as the reference group, those with SARS‐CoV‐2 Delta and SARS‐CoV‐2 Omicron had aHRs for mortality of 2.1 (95% CI, 1.02–4.3; *p* = .04) and 1.9 (95% CI, 0.88–3.9; *p* = .11), respectively (Supporting Information: Tables 2‐3).

### 3.4 Sensitivity analysis

When Influenza A and B were considered as a single group, the cumulative incidence of ICU admission was approximately 5.0% (95% CI, 2.7–7.2) (Supporting Information: Figure 1). Furthermore, in the multivariable Cox proportional hazards model, the risk of ICU admission and mortality were 2.4 (95% CI, [1.4−4.2]; *p* = .002) and 1.2 (95% CI, [0.57−2.4]; *p* = .7), respectively (Supporting Information: Tables 4‐6).

In the matched case‐control analysis (characteristics shown in Table 4) and using patients with influenza B as the reference group, patients with SARS‐CoV‐2 Delta and SARS‐CoV‐2 Omicron had HRs for ICU admission of 3.7 (95% CI: 1.8–7.6; *p* < .001) and 1.5 (95% CI: 0.63–3.6; *p* = .36), respectively, according to the multivariable Cox proportional hazards model (Table 5). Regarding the risk of mortality, patients with SARS‐CoV‐2 Delta and SARS‐CoV‐2 Omicron had HRs of 3.0 (95% CI: 1.7–5.6; *p* < .001) and 2.7 (95% CI: 1.4–5.1; *p* = .002), respectively, in the multivariable Cox proportional hazards model (Table 6).

**Table 4:** Patient characteristics in the age and sex matched patients.

Characteristics			SARS‐CoV‐2 variants		Influenza B (*n* = 267)	Total (*n* = 1459)	*p* Value
			Delta (*n* = 267)	Omicron (*n* = 267)			
Sex, male (*n*%)			142 (53)	143 (54)	130 (49)	415 (52)	.46
Age, median [IQR]			75 [64−83]	74 [61−82]	73 [64−83]	74 [63−83]	.47
Age group		18−44	16 (6.0)	18 (6.7)	18 (6.7)	52 (6.5)	.91
		45−64	55 (21)	65 (24)	56 (21)	176 (22)	
		65−79	104 (39)	100 (38)	98 (37)	302 (38)	
		80≤	92 (35)	84 (32)	95 (36)	271 (34)	
Comorbidities	Diabetes		67 (25)	59 (22)	59 (22)	185 (23)	.64
	Cardiac diseases		146 (55)	138 (52)	157 (59)	441 (55)	.25
	Pulmonary diseases		65 (24)	69 (26)	73 (27)	207 (26)	.73
	Renal diseases		28 (11)	35 (13)	16 (6.0)	79 (9.9)	.02
	Receiving Immunosuppressive agents^a^		31 (12)	38 (14)	5 (1.9)	74 (9.2)	<.001
	Cancer		25 (9.4)	51 (19)	48 (18)	124 (16)	.003
Number of comorbidities	0		63 (24)	73 (27)	77 (29)	213 (27)	.37
	1		101 (38)	94 (35)	95 (36)	290 (36)	
	2		77 (29)	65 (24)	74 (28)	216 (27)	
	3≤		26 (9.7)	35 (13)	21 (7.9)	82 (10)	
Received treatment, Yes (n%)b			184 (69)	155 (58)	195 (73)	534 (67)	<.001
Vaccinated, Yes (*n*%)			159 (59.6)	182 (68.2)	92 (34.5)	433 (54.1)	<.001
ICU‐admission within 30 days			29 (11)	12 (4.5)	11 (4.1)	52 (6.5)	<.001
Death within 30 days			44 (17)	35 (13)	15 (5.6)	94 (12)	<.001
Hospital stay, median [IQR]			4 [2−10]	4 [2−7]	3 [1−6]	4 [1−8]	.004

a The following drugs were considered immunosuppressive: >20 mg prednisolone/daily or equivalent, chemotherapy, biologicals, and methotrexate.

b Dexamethasone, Remdesivir, Monoclonal antibodies, or IL‐6 inhibitors for patients with SARS‐coV‐2 versus Tamiflu for patients with influenza.

**Table 5:** Risk of ICU admission within 30 days after hospitalization in the age and sex matched population.

			Unadjusted model		Multivariable model 1^a^	
			Hazard ratio (95% CI)	*p* Value	Hazard ratio (95% CI)	*p* Value
Viruses	Influenza B		Ref		Ref	‐
	SARS‐CoV2‐Delta		2.8 [1.4; 5.5]	.004	3.7 [1.8; 7.6]	<.001
	SARS‐CoV2‐Omicron		1.1 [0.49; 2.5]	.80	1.5 [0.63; 3.6]	.36
Age (per 10 years increase)			0.99 [0.83; 1.2]	.90	0.84 [0.68; 1.1]	.13
Male sex			4.6 [2.3; 9.5]	<.001		
Vaccinated			0.80 [0.46; 1.4]	.39		
Received treatment			6.3 [2.3; 17]	<.001		
Number of comorbidities		0	Ref	‐		
		1	2.3 [0.93; 5.8]	.07		
		2	2.8 [1.1; 7.1]	.03		
		3≤	4.4 [1.6; 12]	.004		

a Model 1: Due to the low number of patients, it was not possible to stratify model by age group. Therefore, the model was adjusted for age and stratified by sex, number of comorbidities, treatment, and vaccination status.

**Table 6:** Risk of death within 30 days after hospitalization in the age and sex matched population.

			Unadjusted model		Multivariable model 1^a^	
			Hazard ratio (95% CI)	*p* Value	Hazard ratio (95% CI)	*p* Value
Viruses	Influenza B		Ref		Ref	‐
	SARS‐CoV2‐Delta		3.1 [1.7; 5.6]	<.001	3.0 [1.7; 5.6]	<.001
	SARS‐CoV2‐Omicron		2.4 [1.3; 4.3]	.004	2.7 [1.4; 5.1]	.002
Age (per 10 years increase)			1.8 [1.5; 2.3]	<.001	1.9 [1.5; 2.3]	<.001
Male sex			1.4 [0.91; 2.1]	.13		
Vaccinated			1.4 [0.92; 2.1]	0.12		
Received treatment			2.1 [1.2; 3.4]	.005		
Number of comorbidities		0	Ref	‐		‐
		1	1.8 [0.97; 3.1]	.06		‐
		2	1.8 [1.00; 3.4]	.05		‐
		3≤	2.2 [1.1; 4.6]	.03		‐

a Model 1: Due to the low number of patients, it was not possible to stratify model by age group. Therefore, the model was adjusted for age and stratified by sex, number of comorbidities, treatment, and vaccination status.

## 4 DISCUSSION

Our study found that the risks of ICU admission were similar for patients who were hospitalized due to either the SARS‐CoV‐2 Omicron variant or influenza B. However, we found a significantly higher risk of ICU admission for patients who were hospitalized with the SARS‐CoV‐2 Delta variant compared to those hospitalized with influenza B. Furthermore, the risk of all‐cause mortality in patients with SARS‐CoV‐2 Delta or Omicron variants was more than threefold higher than in those with influenza B. When patients with influenza A were used as the reference group, patients with SARS‐CoV‐2 Delta but not Omicron had a higher risk of mortality. These findings are useful for post‐pandemic preparedness, considering that COVID‐19 is becoming an endemic disease and SARS‐CoV‐2 coexists with other respiratory pathogens.

Patients with SARS‐CoV‐2 Delta exhibited the highest cumulative incidence of ICU admissions at day 30, followed by the patients with influenza A, whereas those with SARS‐CoV‐2 Omicron and influenza B exhibited a similar but lower cumulative incidence of ICU admissions. A Swiss study found that patients with SARS‐CoV‐2 Omicron and those with influenza (2018–2022) exhibited a similar cumulative incidence of ICU admissions.^27^ However, another Swiss study that included patients with SARS‐CoV‐2 and influenza before March 2020 when wild‐type SARS‐CoV‐2 was predominant, found that the cumulative incidence of ICU admission for patients with SARS‐CoV‐2 was almost twofold higher than that for patients with influenza.^28^

We observed trends toward higher risks of ICU admission and mortality for patients with influenza A, compared to those with influenza B; however, the differences were not statistically significant, likely due to the limited number of patients with influenza A in this cohort. In adult patients, influenza A can reportedly cause more severe disease than influenza B.^13^ However, the severity of influenza disease varies widely.^29^ We included patients from 2017/18, which was a harsh influenza season worldwide.^30^, ^31^, ^32^ The proportions of circulating subtypes and disease severity in the 2017/18 influenza season differed from previous influenza seasons, with reduced levels of vaccine efficacy and increased levels of hospitalization in Denmark during the winter of 2017/18.^30^ According to the surveillance report by the SSI in Denmark, among more than 5500 patients with influenza in the 2017/2018 season, 68% were affected by influenza B, 17% by influenza A (H3N2), and 14% by influenza A (H1N1)pdm09.^30^ The predominant virus during this season was the Yamagata lineage of influenza B, which uncommonly affects older adults and was not targeted by the seasonal vaccine.^30^ Therefore, our study primarily compared SARS‐CoV‐2 variants with the Yamagata lineage of influenza B. Interestingly, the number of patients affected by the Yamagata lineage of influenza B has significantly declined since the peak of the COVID‐19 pandemic.^33^, ^34^ The reasons for this decline have not been determined but may include an inherent weakness in this lineage that made it less able to flourish during the COVID‐19 pandemic, and a lower effective reproductive number than other lineages.^33^

Regarding the risk of mortality, we found a higher risk for patients hospitalized with SARS‐CoV‐2 Delta compared to influenza A/B, and also a higher risk for patients hospitalized with Omicron compared to influenza B, which aligns with previous studies, even studies that included patients with COVID‐19 from before the Delta and Omicron waves.^35^, ^36^, ^37^ By contrast, a study conducted in Germany observed similar risks of mortality for Omicron and influenza.^38^ This study included patients from various influenza seasons spanning 2018−2022, but it did not ascertain the composition of subtypes, which could explain the contrasting findings.^38^ Different results may also be due to differences among sample sizes, patient characteristics (e.g., sex, age, or comorbidities), or regional differences in vaccination or treatment strategies.^35^, ^36^, ^38^, ^39^, ^40^

In our study, most patients who were hospitalized with either influenza or SARS‐COV‐2 variants were at risk of having severe outcomes because, for example, they had a chronic disease or were older than 65 years. This underlines the importance of protecting these patient groups using vaccination programs. Nevertheless, a large proportion of influenza patients (65%) were not vaccinated before admission. Furthermore, as described above, the trivalent vaccine administered that year was not optimal for the circulating influenza B virus, resulting in reduced effectiveness of this component of the vaccine.^30^ Vaccination rates for SARS‐CoV‐2 were very high in Denmark, and as of May 22, 2022, 90% of the population had received a first dose, 89% had received a second dose, and 76% had received a third dose. Additionally, more than 90% of individuals were vaccinated with an mRNA vaccine.^20^

Our study adds to the existing literature by directly comparing hospitalizations due to SARS‐CoV‐2 Delta and Omicron with those due to influenza A and B, which helps contextualize the differences in disease outcomes. Despite the strengths of our study, it is important to acknowledge some limitations. First, the 2017/18 influenza season was unusual in Denmark and other European countries, as described above. Additionally, the mismatch between the influenza B virus that was targeted by the vaccine during that season and the circulating influenza B strain probably reduced the vaccine's effectiveness.^30^ These factors may have influenced our results, leading to underestimates in the risks of ICU admission and mortality that resulted from SARS‐CoV‐2 Delta and Omicron infections, compared to influenza B infections. Second, the study design was retrospective, and the influenza cohort predated the appearance of SARS‐CoV‐2 Delta and Omicron, which could introduce biases and limitations in data collection. Third, the specific characteristics of the patient population in our study may differ from those in other geographic regions or healthcare settings, affecting the generalizability of our findings. There were differences in patient characteristics; to reduce bias, we conducted an age and sex‐matched sensitivity analysis, which aligned with our main analyses. Forth, early intervention within the first few days of symptom onset can influence the severity of disease and risk of death.^41^, ^42^ However, due to incomplete data on the onset of symptoms, we chose to use treatments as a proxy in the adjusted Cox regression model.

In conclusion, we found that patients hospitalized with influenza B had a similar risk of ICU admission to patients hospitalized with the SARS‐CoV‐2 Omicron variant in this cohort of patients. In contrast, the risk of ICU admission was much higher in patients hospitalized with the historically dominant Delta variant. Nevertheless, the risk of mortality after hospitalization due to each of the SARS‐CoV‐2 variants was more than threefold higher than that due to influenza B. However, only the Delta variant was associated with a significantly higher risk of mortality than influenza A in our cohort of hospitalized patients. Although Delta and Omicron are not the dominant variants at present, our findings highlight the need for continued monitoring, prevention, and intervention strategies to mitigate the impact of severe infections caused by SARS‐CoV‐2 variants and to improve patient outcomes. Furthermore, these results contribute to the existing literature and underscore the importance of ongoing research and surveillance to inform public health strategies for managing viral infections.

## AUTHOR CONTRIBUTIONS

**Omid Rezahosseini, Adin Sejdic, Casper Roed, Mads Frederik Eiberg, Jon Gitz Holler, Zitta Barrella Harboe**: Conceptualization; data curation, methodology, writing—original draft. **Omid Rezahosseini**: Formal analysis. **Zitta Barrella Harboe**: Funding acquisition. **Jon Gitz Holler, Zitta Barrella Harboe**: Supervision. **Lene Nielsen, Jonas Boel, Caroline Klint Johannesen, Maarten van Wijhe, Kristina Træholt Franck, Sisse Rye Ostrowski, Birgitte Lindegaard, Thea K. Fischer, Troels Bygum Knudsen**: Methodology, writing—review and editing, **The COVID‐19 Omicron Delta study group collaborators**: Data collection, Sample analysis, Editing. All co‐authors and collaborators, read, approved, and commented on the manuscript.

## THE COVID‐19 OMICRON DELTA STUDY GROUP COLLABORATORS

Betina Lindgaard‐Jensen, Christian Søborg, Thyge Lynghøj Nielsen, Peter Haahr Bernhard, Emilie Marie Juelstorp Pedersen*, Gertrud Baunbæk Egelund, Inger Hee Mabuza Mathiesen, Naja Zenius Jespersen, Pelle Trier Petersen*, Hans Eric Sebastian Seitz‐Rasmussen, and Barbara Bonnesen Bertelsen (Department of Pulmonary and Infectious Diseases, Copenhagen University Hospital, North Zealand, Hillerød, Denmark); Morten Bestle*, Henrik Andersen, and Thomas Ulrik Skram (Department of Intensive Care, Copenhagen University Hospital, North Zealand, Hillerød, Denmark); Sarah Altaraihi, Pradeesh Sivapalan*, and Jens‐Ulrik Stæhr Jensen* (Department of Medicine, Section of Respiratory Medicine, Copenhagen University Hospital—Herlev and Gentofte Hospital, Copenhagen, Denmark); Kristian Bagge and Kristina Melbardis Jørgensen (Department of Clinical Microbiology, Copenhagen University Hospital, Amager‐Hvidovre Hospital, Denmark); Magnus Glindvad Ahlström (Department of Clinical Microbiology, Copenhagen University Hospital, Herlev‐Gentofte Hospital, Denmark); Sofie Rytter, Nina le Dous, and Pernille Ravn (Department of Internal Medicine, Section for Infectious Diseases, Copenhagen University Hospital, Herlev‐Gentofte Hospital, Denmark); Nanna Reiter (Department of Intensive Care, Copenhagen University Hospital, Bispebjerg‐Frederiksberg Hospital, Denmark); Daria Podlekareva and Jesper Andreas Knudsen (Department of Respiratory Medicine and Infectious Diseases, Copenhagen University Hospital, Bispebjerg‐Frederiksberg Hospital, Denmark); Lars‐Erik Kristensen* (Parker Institut, Copenhagen University Hospital, Bispebjerg‐Frederiksberg Hospital, Denmark); Cæcilie Leding and Thomas Benfield* (Department of Infectious Diseases, Copenhagen University Hospital, Amager‐Hvidovre Hospital, Denmark); Ole Kirk* (Department of Infectious Diseases, Copenhagen University Hospital, Rigshospitalet, Denmark); Sigurdur Thor Sigurdsson (Department of Neurointensive Care, Copenhagen University Hospital, Rigshospitalet, Denmark); Martin Schou Pedersen (Department of Clinical Microbiology, Copenhagen University Hospital, Rigshospitalet, Denmark).

*These authors have a secondary affiliation to Department of Clinical Medicine, University of Copenhagen, Copenhagen, Denmark.

## CONFLICTS OF INTEREST STATEMENT

O. R. received a grant from Rigshospitalet and a grant from A. P. Møller Fonden not related to this work. Mads Ejberg has received research funding from the Danish Cancer Society (grant number KBVU‐M. S. R320‐A18526). K. T. F. has received grants from the Lundbeck Foundation (grant number R349‐2020‐835). Z. B. H. received grants from the Independent Research Fund (grant number 0134‐00257B), the Lundbeck Foundation (grant number R349‐2020‐835), the Helen Rudes Foundation, and the Danish Cancer Society (grant number KBVU‐MS R327‐A19137).

## ETHICS STATEMENT

The study was approved by the Danish Health and Medicines Authority (ID: 31‐1521‐263) and the Danish Data Protection Agency (P‐2020‐375). Informed consent is not required for studies using data from health registers in Denmark.

## Supporting information

Supporting information.

Supporting information.

## Data Availability

The data supporting this study's findings are available from the corresponding author upon reasonable request. The data are not publicly available due to Danish legislation.
