# Supplementary material for: Risk of intensive care unit admission and mortality in patients hospitalized due to influenza A or B and SARS‑CoV‑2 variants Omicron or Delta
Source: Immun Inflamm Dis. 2024 Jul 5;12(7):e1269. doi: 10.1002/iid3.1269 (PMC11225085; doi:10.1002/iid3.1269)
Supplement: Supplementary file 1 — Supporting information. [file IID3-12-e1269-s002.docx]

**Supplementary Tables**

Supplementary Table 1: The prescribed medicines to the patients with SARS-CoV-2 variants

| Variable | | SARS-CoV-2 Delta (n=717) | SARS-CoV-2 Omicron (n=381) | Total (n=1,098) | p-value |
| --- | --- | --- | --- | --- | --- |
| Number of the drugs | 1 | 125 (18) | 73 (19) | 198 (18) | < 0.001 |
|  | 2 | 320 (45) | 114 (30) | 434 (40) |  |
|  | 3 | 45 (6.3) | 16 (4.3) | 61 (5.6) |  |
| Received Dexamethasone (Yes, n(%)) | | 449 (63) | 160 (42) | 609 (56) | < 0.001 |
| Received monoclonal antibodies (Yes, n(%)) | | 92 (13) | 42 (11) | 134 (12) | 0.45 |
| Received IL-6 inhibitors (Yes, n(%)) | | 46 (6.5) | 9 (2.4) | 55 (5.0) | 0.005 |
| Received Remdisivir (Yes, n(%)) | | 362 (51) | 152 (40) | 514 (47) | < 0.001 |

**Analyses when influenza A was considered as the reference**

Supplementary Table 2: Risk of ICU admission within 30 days after hospitalization using influenza A as the reference

|  | | Univariable model | | Multivariable model* | |
| --- | --- | --- | --- | --- | --- |
|  |  | Hazard Ratio (95% CI) | p-value | Hazard Ratio (95% CI) | p-value |
| Viruses | Influenza A | Ref | - | Ref | - |
|  | Influenza B | 0.54 [0.21;1.4] | 0.21 | 0.48 [0.18;1.3] | 0.14 |
|  | SARS-CoV2-Delta | 1.4 [0.66;3.1] | 0.36 | 1.5 [0.65;3.3] | 0.36 |
|  | SARS-CoV2-Omicron | 0.52 [0.21;1.3] | 0.16 | 0.71 [0.28;1.8] | 0.48 |
| Age group (years) | 18-44 | Ref | - | - | - |
|  | 45-64 | 1.7 [0.90;3.2] | 0.10 | - | - |
|  | 65-79 | 1.92 [1.06;3.5] | 0.03 | - | - |
|  | 80≤ | 0.60 [0.29;1.3] | 0.18 | - | - |
| Male sex | | 2.7 [1.8;4.1] | <0.001 | - | - |
| Vaccinated | | 0.70 [0.48;1.03] | 0.07 | - | - |
| Received at least one treatment** | | 13 [5.3;32] | <0.001 | - | - |
| Number of comorbidities | 0 | Ref | - | - | - |
|  | 1 | 1.3 [0.80;2.1] | 0.30 | - | - |
|  | 2 | 1.36 [0.81;2.3] | 0.24 | - | - |
|  | 3≤ | 1.7 [0.86;3.3] | 0.13 | - | - |
| *Model: Stratified by age group, sex, vaccination status, Tx, and number of comorbidities  ****Dexamethasone, remdesivir, monoclonal antibodies, and il6-inhibitors for patients with SARS-coV-2 vs. Oseltamivir for patients with influenza | | | | | |

Supplementary Table 3: Risk of death within 30 days after hospitalization using influenza A as the reference

|  | | Univariable model | | Multivariable model* | |
| --- | --- | --- | --- | --- | --- |
|  |  | Hazard Ratio (95% CI) | p-value | Hazard Ratio (95% CI) | p-value |
| Viruses | Influenza A | Ref | - | Ref | - |
|  | Influenza B | 0.57 [0.25;1.3] | 0.19 | 0.60 [0.24;1.3] | 0.18 |
|  | SARS-CoV2-Delta | 1.5 [0.75;2.9] | 0.26 | 2.1 [1.02;4.3] | 0.04 |
|  | SARS-CoV2-Omicron | 1.3 [0.66;2.7] | 0.42 | 1.9 [0.88;3.9] | 0.11 |
| Age group (years) | 18-44 | Ref | - | - | - |
|  | 45-64 | 2.3 [0.61;8.3] | 0.23 | - | - |
|  | 65-79 | 12 [3.8;38] | <0.001 | - | - |
|  | 80≤ | 21 [6.8;67] | <0.001 | - | - |
| Male sex | | 1.3 [0.94;1.7] | 0.12 | - | - |
| Vaccinated (Yes) | | 1.3 [0.95;1.7] | 0.10 | - | - |
| Received at least one treatment** | | 2.2 [1.5;3.2] | <0.001 | - | - |
| Number of comorbidities | 0 | Ref | - | - | - |
|  | 1 | 1.8 [1.2;2.8] | 0.007 | - | - |
|  | 2 | 2.4 [1.6;3.8] | <0.001 | - | - |
|  | 3≤ | 3.1 [1.8;5.2] | <0.001 | - | - |
| *Model: Stratified by age group, sex, vaccination status, treatment, and number of comorbidities  ** Dexamethasone, remdesivir, monoclonal antibodies, and il6-inhibitors for patients with SARS-coV-2 vs. Oseltamivir for patients with influenza | | | | | |

**Analyses when Influenza A and B were considered as a single group**

Supplementary Table 4: Patient characteristics in the total population using influenza A and B as a single group

| Characteristics | | SARS-CoV-2 variants | | Influenza A/B (n=361) | Total (n=1459) | p-value |
| --- | --- | --- | --- | --- | --- | --- |
|  |  | Delta (n=717) | Omicron (n=381) |  |  |  |
| Sex, Male (n%) | | 398 (56) | 184 (48) | 184 (51) | 766 (53) | 0.05 |
| Age, median [IQR] | | 69 [51, 80] | 72 [50, 83] | 73 [63, 83] | 72 [54, 81] | < 0.001 |
| Age group | 18-44 | 137 (19) | 82 (22) | 27 (7.5) | 246 (17) | < 0.001 |
|  | 45-64 | 188 (26) | 67 (18) | 77 (21) | 332 (23) |  |
|  | 65-79 | 218 (30) | 120 (32) | 128 (36) | 466 (32) |  |
|  | 80≤ | 174 (24) | 112 (29) | 129 (36) | 415 (28) |  |
| Comorbidities | Diabetes | 150 (21) | 71 (19) | 77 (21) | 298 (20) | 0.6 |
|  | Cardiac diseases | 312 (44) | 171 (45) | 210 (58) | 693 (48) | < 0.001 |
|  | Pulmonary diseases | 166 (23) | 91 (24) | 106 (30) | 363 (25) | 0.07 |
|  | Renal diseases | 61 (8.5) | 43 (11.3) | 21 (5.8) | 125 (8.6) | 0.03 |
|  | Receiving Immunosupprive agents* | 77 (11) | 50 (13) | 12 (3.3) | 139 (9.5) | < 0.001 |
|  | Cancer | 66 (9.2) | 60 (16) | 68 (19) | 194 (13) | < 0.001 |
| Number of comorbidities | 0 | 253 (35) | 124 (33) | 101 (28) | 478 (33) | < 0.001 |
|  | 1 | 233 (33) | 135 (35) | 126 (35) | 494 (34) |  |
|  | 2 | 167 (23) | 83 (22) | 107 (30) | 357 (25) |  |
|  | 3≤ | 64 (8.9) | 39 (10) | 27 (7.5) | 130 (8.9) |  |
| Received treatment, Yes (n%)** | | 490 (68) | 203 (53) | 270 (75) | 963 (66) | < 0.001 |
| Vaccinated, Yes (n%) | | 334 (47) | 257 (68) | 127 (35) | 718 (49) | < 0.001 |
| ICU admission within 30 days | | 75 (11) | 15 (3.9) | 18 (5.0) | 108 (7.4) | < 0.001 |
| Death within 30 days | | 99 (14) | 48 (13) | 24 (6.6) | 171 (12) | 0.002 |
| Hospital stay, median [IQR] | | 4 [2, 9] | 3 [1, 6] | 3 [1, 7] | 4 [1, 8] | 0.002 |
| *Following drugs were considered treatment:  Dexamethasone, Remdesivir, Monoclonal antibodies, or IL-6 inhibitors for patients with SARS-coV-2 vs. Tamiflu for patients with influenza | | | | | | |

Supplementary Table 5: Risk of ICU admission within 30 days after hospitalization using influenza A/B as a single group as the reference

|  | | | | Unadjusted model | | Adjusted model* | |
| --- | --- | --- | --- | --- | --- | --- | --- |
|  |  |  |  | Hazard Ratio (95% CI) | p-value | Hazard Ratio (95% CI) | p-value |
| Viruses | Influenza | | | Ref |  | Ref | - |
|  | SARS-CoV2-Delta | | | 2.2 [1.30;3.6] | 0.003 | 2.4 [1.4;4.2] | 0.002 |
|  | SARS-CoV2-Omicron | | | 0.79 [0.40;1.6] | 0.51 | 1.2 [0.57;2.4] | 0.7 |
| Age group (years) | | | 18-44 | Ref | - |  |  |
|  |  |  | 45-64 | 1.7 [0.90;3.2] | 0.1 |  |  |
|  |  |  | 65-79 | 1.9 [1.1;3.5] | 0.03 |  |  |
|  |  |  | 80≤ | 0.60 [0.29;1.3] | 0.18 |  |  |
| Male sex | | | | 2.7 [1.8;4.1] | <0.001 |  |  |
| Vaccinated | | | | 0.70 [0.48;1.03] | 0.07 |  |  |
| Received treatment | | | | 12 [5.0;30] | <0.001 |  |  |
| Number of comorbidities | | 0 | | Ref | - |  |  |
|  |  | 1 | | 1.2 [0.77;2.0] | 0.38 |  |  |
|  |  | 2 | | 1.3 [0.76;2.1] | 0.35 |  |  |
|  |  | 3≤ | | 1.5 [0.78;3.0] | 0.22 |  |  |
| * stratified by age group, sex, number of comorbidities, treatment, and vaccination status | | | | | | | |

Supplementary Table 6: Risk of death within 30 days after hospitalization using influenza A/B as a single group as the reference

|  | | | | Unadjusted model | | Adjusted model* | |
| --- | --- | --- | --- | --- | --- | --- | --- |
|  |  |  |  | Hazard Ratio (95% CI) | p-value | Hazard Ratio (95% CI) | p-value |
| Viruses | Influenza | | | Ref | - | Ref | - |
|  | SARS-CoV2-Delta | | | 2.2 [1.4;3.4] | <0.001 | 3.2 [2.0;5.1] | <0.001 |
|  | SARS-CoV2-Omicron | | | 2.0 [1.2;3.2] | 0.007 | 2.8 [1.7;4.7] | <0.001 |
| Age group (years) | | | 18-44 | Ref | - |  | - |
|  |  |  | 45-64 | 2.3 [0.61;8.3] | 0.23 |  |  |
|  |  |  | 65-79 | 12 [3.8;38] | <0.001 |  |  |
|  |  |  | 80≤ | 21 [6.7;67] | <0.001 |  |  |
| Male sex | | | | 1.3 [0.93;1.7] | 0.12 |  |  |
| Vaccinated | | | | 1.3 [0.94;1.7] | 0.10 |  |  |
| Received treatment | | | | 2.2 [1.5;3.2] |  |  |  |
| Number of comorbidities | | 0 | | Ref | - |  |  |
|  |  | 1 | | 1.80 [1.2;2.8] | 0.007 |  |  |
|  |  | 2 | | 2.4 [1.6;3.8] | <0.001 |  |  |
|  |  | 3≤ | | 3.1 [1.8;5.2] | <0.001 |  |  |
| * stratified by age group, sex, number of comorbidities, treatment, and vaccination status | | | | | | | |


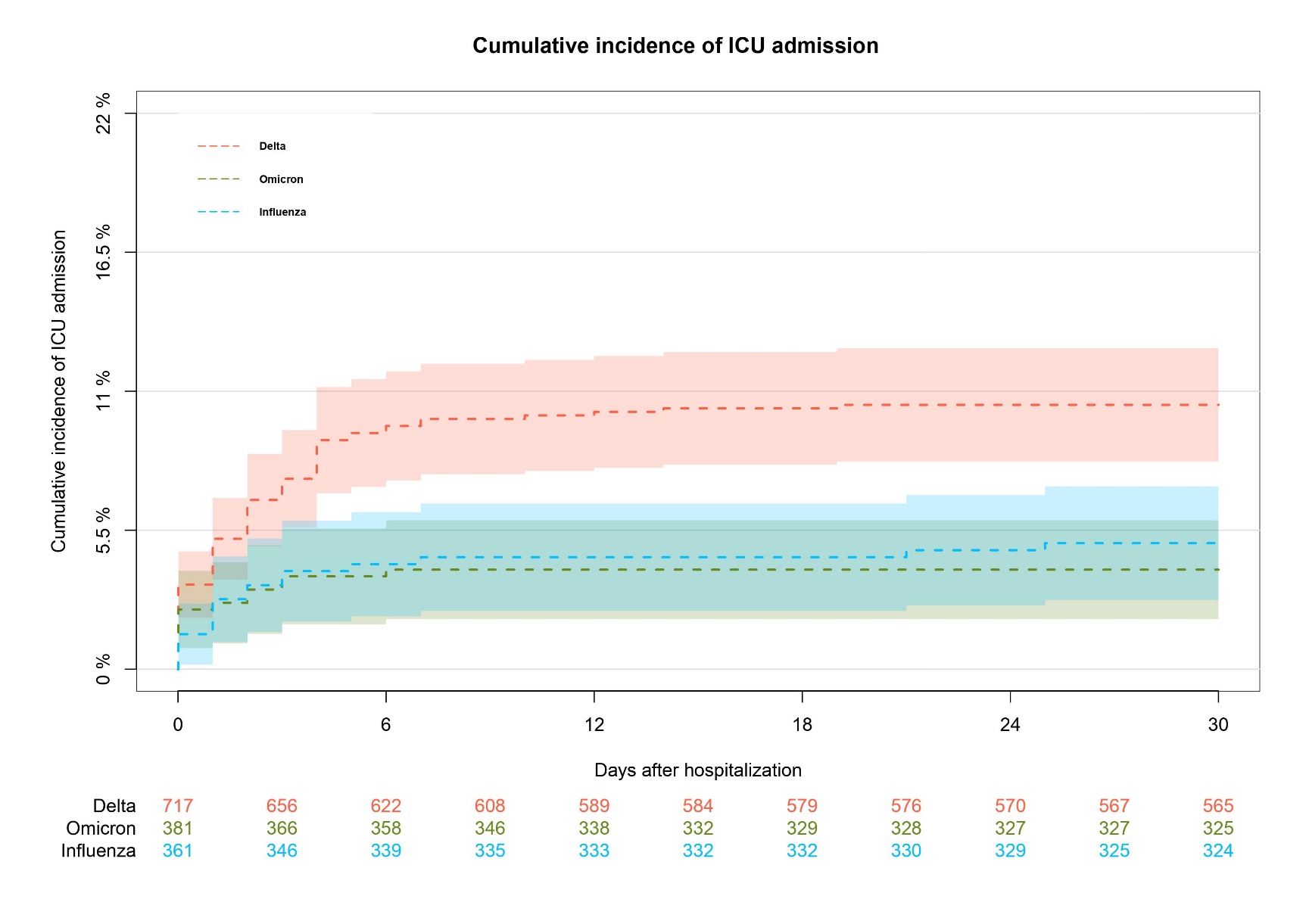


Supplementary Figure 1: The cumulative incidence of ICU admission at day 30 after hospitalization was 11% (8.2-13), 4.0% (2.0-5.9), and 5.0% (2.7-7.2) for patients with SARS-CoV-2 delta, SARS-CoV-2 omicron, and influenza infections, respectively (p < 0.001),
