# Supplementary material for: Risk of intensive care unit admission and mortality in patients hospitalized due to influenza A or B and SARS‑CoV‑2 variants Omicron or Delta
Source: Immun Inflamm Dis. 2024 Jul 5;12(7):e1269. doi: 10.1002/iid3.1269 (PMC11225085; doi:10.1002/iid3.1269)
Supplement: Supplementary file 2 — Supporting information. [file IID3-12-e1269-s001.docx]

Dear Editor,

Thank you for the opportunity to revise and improve our manuscript. Below, we provide a point-by-point response to your comments. All references to line and page numbers correspond to the manuscript version with "Track Changes".

Additionally, we would like to inform you that, for further clarification, we have transferred a portion of the material initially reported as supplementary into the main text which can be found on:

**Page 7**, lines 11-21; **Page 8**, lines 2-10 and lines 20-22; **Page 9**, lines 1-2 and 10-13; **Page 10**, lines 8-11 and 18-21; **Page 11**, 9-13 and 18-22; **Page 12**, lines 1-8, **Pages 29-21**, Tables 4-6.

Comments to the Author

Thank you for the invitation to review this manuscript. I read with interest the data reported by our colleagues. Their experience in caring for patients with influenza and COVID-19 is valuable. The aim of the study is to quantify the risk of ICU admission and mortality in patients hospitalized for influenza or COVID-19 (SARS-CoV-2 variants Delta and Omicron). Two cohorts of adult patients from different periods 2017/18 for influenza and 2021/22 for COVID-19 are presented. There are a number of issues I would like to highlight about this manuscript:

**Response to the Reviewer:**

We appreciate your time and the comments.

Comment 1:

- The inclusion criteria are not clear. Were all consecutive patients who did not have any of the exclusion criteria enrolled? This needs to be clarified.

Response to Comment 1:

As you correctly noted, we included all patients who met none of the exclusion criteria. For further clarification about our inclusion criteria, it was necessary to have more details about the two cohorts. The details were reported as supplementary methods. Now, we have transferred them into the main text. It can be found on page 7, lines 11-21, and page 8, lines 2-10 which read:

” The first cohort was from a single center and comprised inpatients hospitalized due to influenza A/B from 1 October 2017 to 1 April 2018 (6 months) at Nordsjællands Hospital (NOH) (Supplementary Methods). The second cohort included patients hospitalized with SARS-CoV-2 variants Delta and Omicron between 1 September 2021 and 11 February 2022, at several university hospitals (including NOH, Bispebjerg, Rigshospitalet, Hvidovre, Herlev, and Bornholm) (Supplementary Methods).”

It now reads:

” The cohort of patients with influenza A/B

Nordsjællands Hospital (NOH) is one of the university hospitals in the Capital Region, which provides nonstop medical services to approximately 323,000 inhabitants in North Zealand. The NOH emergency department evaluates/treats approximately 85,000 patients annually. All symptomatic patients were screened for influenza at the emergency department and had nasopharyngeal swabs collected for analysis via real-time polymerase chain reaction (RT-PCR) using the Prodesse ProFlu™+ (Hologic, Inc., Marlborough, MA, USA) assay procedure to identify the influenza A/B viruses and Respiratory Syncytial Virus. Patients with a positive RT-PCR test result for influenza A or influenza B were distinguished by further testing at the Department of Clinical Microbiology at Herlev Hospital, Denmark. The administrative database at NOH was cross-checked to confirm the hospitalizations.

The cohort of patients with SARS-CoV-2 variants Delta and Omicron

In Denmark, Delta was the predominant SARS-CoV-2 variant from July to December 2021, with the first cases of the Omicron variant detected in November 2021 [1,2]. Omicron cases subsequently peaked in mid-January 2022 [1,2]. Therefore, all individuals who tested positive for SARS-CoV-2 via RT-PCR and had a hospital contact between 1 September 2021 and 11 February 2022 were eligible for inclusion. The cohort characteristics and surveillance and screening algorithms for SARS-CoV-2 variants in Denmark have been described previously [3]. Variant identification relied on whole-genome sequencing or variant-specific RT-PCR conducted at the Statens Serum Institut or departments of clinical microbiology within the region.”

Furthermore, we added on page 8, line 19, which reads:

” All patients who did not meet the exclusion criteria were included.”

Comment 2:

- According to Table 1 there were differences between patient characteristics according to age/age group, comorbidities. This may be biased when making comparisons between the two viruses (with their subtypes). The distribution of these comorbidities in relation to the age and sex of the patients is important and may significantly influence the outcome. The most appropriate design for such an analysis was a case-control design, matching patients with influenza and COVID-19 by age, sex and comorbidities. In this way the results generated showed the real impact of the two infections.

Response to Comment 2:

We appreciate your comment. Due to the limited number of patients with influenza A, the matching process proved unsatisfactory. Consequently, we opted to report on the entire cohort. However, we conducted a sensitivity analysis excluding patients with influenza A. In this analysis, we carried out a matched case-control study using Influenza B as the reference. The findings were detailed in the supplementary material. For enhanced clarity, we have moved methods and results related to the case-control study from supplementary material to the main manuscript, which reads:

Page 11, lines 21-22, page 12, lines 1-8:

” Furthermore, we conducted a matched case-control analysis to compare the risk of ICU admission and mortality 30 days after hospitalization. Because of the limited number of patients with influenza A, the matching process was unsatisfactory; hence, we decided to exclude patients with influenza A. Therefore, cases were defined as patients with influenza B, whereas controls were patients with SARS-CoV-2 variants Omicron or Delta. Cases and controls were matched based on age and sex using the MatchIt package in R software (ver. 4.3.1; R Development Core Team, 2023), and the nearest neighbor matching method was used with a 1:1 ratio. It was not possible to stratify for all age groups in the matched analyses for the multivariable model. Therefore, the model was stratified by sex, number of comorbidities, treatment, and vaccination status and adjusted for age (per 10-year increase).”

Furthermore, we added a new subheading to the results, and revised in the results accordingly. The new subheading is called “Sensitivity analysis”, page 14, lines 1-13, which reads:

“Sensitivity analysis

When Influenza A and B were considered as a single group, the cumulative incidence of ICU admission was approximately 5.0% (95% CI, 2.7–7.2) (Supplementary Figure 1). Furthermore, in the multivariable Cox proportional hazards model, the risk of ICU admission and mortality were 2.4 (95% CI, [1.4;4.2]; p= 0.002) and 1.2 (95% CI, [0.57;2.4]; p=0.7), respectively (Supplementary Tables 4-6).

In the matched case-control analysis (characteristics shown in Table 4) and using patients with influenza B as the reference group, patients with SARS-CoV-2 Delta and SARS-CoV-2 Omicron had HRs for ICU admission of 3.7 (95% CI: 1.8–7.6; p < 0.001) and 1.5 (95% CI: 0.63–3.6; p = 0.36), respectively, according to the multivariable Cox proportional hazards model (Table 5). Regarding the risk of mortality, patients with SARS-CoV-2 Delta and SARS-CoV-2 Omicron had HRs of 3.0 (95% CI: 1.7–5.6; p < 0.001) and 2.7 (95% CI: 1.4–5.1; p = 0.002), respectively, in the multivariable Cox proportional hazards model (Table 6).”

We also added in the limitations, on page 17, line 21-23, and page 18, lines 1-2 which read:

“Third, the specific characteristics of the patient population in our study may differ from those in other geographic regions or healthcare settings, affecting the generalizability of our findings. “

It now reads:

“Third, the specific characteristics of the patient population in our study may differ from those in other geographic regions or healthcare settings, affecting the generalizability of our findings. There were differences in patient characteristics; to reduce bias, we conducted an age and sex-matched sensitivity analysis, which aligned with our main analyses.”

The following tables moved from supplementary material to the main text on pages 29-31:

Table 4: Patient characteristics in the age and sex matched patients

| Characteristics | | | SARS-CoV-2 variants | | Influenza B  (n=267) | Total (n=1459) | p-value |
| --- | --- | --- | --- | --- | --- | --- | --- |
|  |  |  | Delta (n=267) | Omicron (n=267) |  |  |  |
| Sex, Male (n%) | | | 142 (53) | 143 (54) | 130 (49) | 415 (52) | 0.46 |
| Age, median [IQR] | | | 75 [64, 83] | 74 [61, 82] | 73 [64, 83] | 74 [63, 83] | 0.47 |
| Age group | | 18-44 | 16 (6.0) | 18 (6.7) | 18 (6.7) | 52 (6.5) | 0.91 |
|  |  | 45-64 | 55 (21) | 65 (24) | 56 (21) | 176 (22) |  |
|  |  | 65-79 | 104 (39) | 100 (38) | 98 (37) | 302 (38) |  |
|  |  | 80≤ | 92 (35) | 84 (32) | 95 (36) | 271 (34) |  |
| Comorbidities | Diabetes | | 67 (25) | 59 (22) | 59 (22) | 185 (23) | 0.64 |
|  | Cardiac diseases | | 146 (55) | 138 (52) | 157 (59) | 441 (55) | 0.25 |
|  | Pulmonary diseases | | 65 (24) | 69 (26) | 73 (27) | 207 (26) | 0.73 |
|  | Renal diseases | | 28 (11) | 35 (13) | 16 (6.0) | 79 (9.9) | 0.02 |
|  | Receiving Immunosuppressive agents* | | 31 (12) | 38 (14) | 5 (1.9) | 74 (9.2) | < 0.001 |
|  | Cancer | | 25 (9.4) | 51 (19) | 48 (18) | 124 (16) | 0.003 |
| Number of comorbidities | 0 | | 63 (24) | 73 (27) | 77 (29) | 213 (27) | 0.37 |
|  | 1 | | 101 (38) | 94 (35) | 95 (36) | 290 (36) |  |
|  | 2 | | 77 (29) | 65 (24) | 74 (28) | 216 (27) |  |
|  | 3≤ | | 26 (9.7) | 35 (13) | 21 (7.9) | 82 (10) |  |
| Received treatment, Yes (n%)** | | | 184 (69) | 155 (58) | 195 (73) | 534 (67) | < 0.001 |
| Vaccinated, Yes (n%) | | | 159 (59.6) | 182 (68.2) | 92 (34.5) | 433 (54.1) | < 0.001 |
| ICU-admission within 30 days | | | 29 (11) | 12 (4.5) | 11 (4.1) | 52 (6.5) | < 0.001 |
| Death within 30 days | | | 44 (17) | 35 (13) | 15 (5.6) | 94 (12) | < 0.001 |
| Hospital stay, median [IQR] | | | 4 [2, 10] | 4 [2, 7] | 3 [1, 6] | 4 [1, 8] | 0.004 |
| *Following drugs were considered as immunosuppressive:  Dexamethasone, Remdesivir, Monoclonal antibodies, or IL-6 inhibitors for patients with SARS-coV-2 vs. Tamiflu for patients with influenza | | | | | | | |

Table 5: Risk of ICU admission within 30 days after hospitalization in the age and sex matched population

|  | | | Unadjusted model | | Multivariable model 1* | |
| --- | --- | --- | --- | --- | --- | --- |
|  |  |  | Hazard Ratio (95% CI) | p-value | Hazard Ratio (95% CI) | p-value |
| Viruses | Influenza B | | Ref |  | Ref | - |
|  | SARS-CoV2-Delta | | 2.8 [1.4;5.5] | 0.004 | 3.7 [1.8;7.6] | <0.001 |
|  | SARS-CoV2-Omicron | | 1.1 [0.49;2.5] | 0.80 | 1.5 [0.63;3.6] | 0.36 |
| Age (per 10 years increase) | | | 0.99 [0.83;1.2] | 0.90 | 0.84 [0.68;1.1] | 0.13 |
| Male sex | | | 4.6 [2.3;9.5] | <0.001 |  |  |
| Vaccinated | | | 0.80 [0.46;1.4] | 0.39 |  |  |
| Received treatment | | | 6.3 [2.3;17] | <0.001 |  |  |
| Number of comorbidities | | 0 | Ref | - |  |  |
|  |  | 1 | 2.3 [0.93;5.8] | 0.07 |  |  |
|  |  | 2 | 2.8 [1.1;7.1] | 0.03 |  |  |
|  |  | 3≤ | 4.4 [1.6;12] | 0.004 |  |  |
| * Model 1: Due to the low number of patients, it was not possible to stratify model by age group. Therefore, the model was adjusted for age and stratified by sex, number of comorbidities, treatment, and vaccination status | | | | | | |

Table 6: Risk of death within 30 days after hospitalization in the age and sex matched population

|  | | | Unadjusted model | | Multivariable model 1* | |
| --- | --- | --- | --- | --- | --- | --- |
|  |  |  | Hazard Ratio (95% CI) | p-value | Hazard Ratio (95% CI) | p-value |
| Viruses | Influenza B | | Ref |  | Ref | - |
|  | SARS-CoV2-Delta | | 3.1 [1.7;5.6] | <0.001 | 3.0 [1.7;5.6] | <0.001 |
|  | SARS-CoV2-Omicron | | 2.4 [1.3;4.3] | 0.004 | 2.7 [1.4;5.1] | 0.002 |
| Age (per 10 years increase) | | | 1.8 [1.5;2.3] | <0.001 | 1.9 [1.5;2.3] | <0.001 |
| Male sex | | | 1.4 [0.91;2.1] | 0.13 |  |  |
| Vaccinated | | | 1.4 [0.92;2.1] | 0.12 |  |  |
| Received treatment | | | 2.1 [1.2;3.4] | 0.005 |  |  |
| Number of comorbidities | | 0 | Ref | - |  | - |
|  |  | 1 | 1.8 [0.97;3.1] | 0.06 |  | - |
|  |  | 2 | 1.8 [1.00;3.4] | 0.05 |  | - |
|  |  | 3≤ | 2.2 [1.1;4.6] | 0.03 |  | - |
| * Model 1: Due to the low number of patients, it was not possible to stratify model by age group. Therefore, the model was adjusted for age and stratified by sex, number of comorbidities, treatment, and vaccination status | | | | | | |

Comment 3:

- At the same time, the inclusion of patients with a positive test in the last 14 days may also be biased, as early intervention in a patient may reduce the risk of mortality compared to a patient in whom management was delayed. Again, grouping of cases according to this aspect should be considered.

Response to Comment 3:

As you correctly noted, early intervention can significantly reduce severity and mortality risk. It has been reported that the interventions are most effective if applied from the onset of symptoms, typically a few days before a PCR test is conducted. Unfortunately, our data on the exact start date of symptoms was incomplete. However, we included treatments in the adjusted Cox regression model.

We added this into our limitations and provided 2 new references, on page 18, lines 2-5, which read:

” Forth, early intervention within the first few days of symptom onset can influence the severity of disease and risk of death [41,42]. However, due to incomplete data on the onset of symptoms, we chose to use treatments as a proxy in the adjusted Cox regression model. “

[41] Godoy P, et al. Effectiveness of Influenza Vaccination and Early Antiviral Treatment in Reducing Pneumonia Risk in Severe Influenza Cases. Vaccines 2024;12.

[42] Mehta RM, et al. A shorter symptom onset to remdesivir treatment (SORT) interval is associated with a lower mortality in moderate-to-severe COVID-19: A real-world analysis. Int J Infect Dis IJID Off Publ Int Soc Infect Dis 2021;106:71–7.

Comment 4:

- Also, how was the follow-up of patients done at 30 days if the study is retrospective and according to table 1 the median length of hospital stay was 3-4 days.

Response to Comment 4:

Thank you for your comment. In Denmark each person has a unique personal number (CPR-number) and it is possible to follow patients from birth to death, using national databases. We described the databases in the supplementary material, it has been moved to the main methods and elaborated on page 10, lines 2-11 which reads:

” Data sources

We obtained data from various sources, including the Danish Civil Register [23], the National Patient Register [24], the Danish Vaccination Register [25], the Danish Microbiology Database (MIBA) [26], and the National COVID-19 surveillance system at the Statens Serum Institut (Copenhagen, Denmark) [21,22]. In Denmark, each person is assigned a unique personal identification number, known as the CPR number. This number enables access to information about an individual's birth date, gender, hospitalization records, and death. It also facilitates tracking patients through the national databases mentioned. Electronic medical records were also reviewed for all unique patients who met the inclusion criteria. Experienced medical doctors and nurses conducted the review process. All extracted information was recorded in a Research Electronic Data Capture (REDCap) database.”

Comment 5:

A number of other small clarifications and revisions would be needed, especially in the discussion.

Response to Comment 5:

We would be happy to make revisions if there are any unclear points in the discussion. Please highlight any areas that require clarification.
